# United States Postal Laws and Our Subscribers

**Published:** 1880-06

**Authors:** 


### UNITED STATES POSTAL LAWS AND OUR SUBSCRIBERS.

THE near approach of the close of the present volume of the JOURNAL leads us to direct attention to the law regulating the relations between publisher and subscriber. Delinquents will find in the perusal of this law profitable suggestions as to their duty, from which we hope to receive some evidence in the form of remittances. Many on our list having failed to notice the polite reminder in the form of a bill enclosed in a previous number, we take the liberty to repeat the hint, and therefore send with this JOURNAL our bills to all subscribers who are in arrears. We need not again assure our readers that while we strive to furnish a superior periodical, we hope their appreciation will be manifested in prompt payment of their subscription.

1. A postmaster is required to give notice *by letter* (returning a paper does not answer the law) when a subscriber does not take his paper out of the office, and state the reasons for its not being taken. Any neglect to do so makes the postmaster *responsible* to the publishers for payment.

2. Any person who takes a paper from the post office, whether directed to his name or another, or whether he has subscribed or not, is responsible for the pay.

3. If a person orders his paper discontinued, he must pay all arrearages, or the publisher may continue to send it until payment is made, and collect the whole amount, *whether it be taken from the office or not.* There can be no legal discontinuance until the payment is made.

4. If the subscriber orders his paper to be stopped at a certain time, and the publisher continues to send, the subscriber is bound to pay for it *if he takes it out of the post office.* The law proceeds upon the ground that a man must pay for what he uses.

5. The courts have decided that refusing to take a newspaper and periodical from the post office, or removing and leaving them uncalled for, is *prima facie* evidence of intentional fraud.

